# Factors influencing preterm infant microbiota and their role in wheezing development

**DOI:** 10.1038/s41390-025-04569-x

**Published:** 2025-11-15

**Authors:** Raúl Cabrera-Rubio, Sonia Alcolea, Laura Sánchez-G-arcía, Patricia Alonso, Leticia Labanda, María Arroyas, Sergio Quevedo, Jorge Atucha, Inmaculada Casas, Francisco Pozo, María Luz García-G-arcía, M. Carmen Collado, Cristina Calvo

**Affiliations:** 1 Department of Biotechnology, Institute of Agrochemistry and Food Technology–National Research Council (IATA-CSIC), Paterna, Valencia, Spain; 2 Pediatric Infectious Diseases Department, Hospital Universitario La Paz, Fundación IdiPaz, Madrid, Spain; 3 CIBER Enfermedades Infecciosas (CIBERINFEC), Madrid, Spain; 4 Neonatology Department, Hospital Universitario La Paz, Madrid, Spain; 5 Neonatology Department, Hospital Severo Ochoa, Leganés, Madrid, Spain; 6 Respiratory Viruses and Influenza Unit at the National Centre for Microbiology ISCIII (CIBERESP ISCIII), Madrid, Spain; 7 Pediatrics Department, Hospital Severo Ochoa, Leganés, Madrid, Spain; 8 Translational Research Network in Pediatric Infectious Diseases (RITIP), Madrid, Spain

## Abstract

**Background:**

This multicenter prospective study, conducted between 2019 and 2022 in two neonatal intensive care units (NICUs) in Madrid (H. Severo Ochoa and H. La Paz), investigated the relationship between nasopharyngeal and gut microbiota in very preterm infants born at <32 weeks of gestation age and the development of recurrent wheezing during the first year of life.

**Methods:**

A total of 91 preterm neonates were enrolled, excluding those with major malformations, genetic disorders, or immunodeficiency. During hospitalization, weekly nasopharyngeal aspirates (NPAs) were collected, beginning in the first 7 days of life. Respiratory viruses were detected via PCR. Stool samples for microbiota were obtained only one time during the first week of life. Microbial composition was characterized through 16S rRNA gene sequencing. The analysis of associations with wheezing specifically included microbiota data from samples collected during the first week of life (stools and NPAs). Microbial profiles were analyzed using bioinformatic and statistical tools, including alpha and beta diversity metrics, redundancy analysis (RDA), and random forest predictive models. Wheezing was defined as **≥**2 episodes of physician-confirmed wheezing requiring medical attention during the first year of life, as reported by caregivers and verified by clinical records.

**Results:**

The results showed that clinical factors such as delivery mode, antibiotic use, type of feeding, and mechanical ventilation significantly influenced microbial profiles. Infants who developed wheezing had a higher abundance of pathogens such as *Klebsiella*, *Escherichia/Shigella*, and *Stenotrophomonas*, whereas *Bifidobacterium* and *Staphylococcus* were more frequent in non-wheezing infants. Both nasopharyngeal and gut microbiota were significantly associated with respiratory outcomes, including hospital admissions and chronic respiratory treatments. Early-life dysbiosis—shaped by antibiotics and artificial feeding—was linked to heightened inflammation and increased risk of respiratory morbidity.

**Conclusions:**

This study suggests that microbial composition during the first week of life can serve as an early predictor of wheezing in preterm infants. Targeted interventions, such as promoting breastfeeding and reducing unnecessary antibiotic use, may help preserve microbial diversity and improve long-term respiratory health in this vulnerable population.

**Impact:**

- The microbiota of preterm neonates during the first week of life plays a pivotal role in determining the risk of respiratory diseases, such as wheezing, later in life.
- Clinical factors such as antibiotic use, delivery mode, and breastfeeding have a profound impact on microbiota composition, with specific genera such as *Moraxella, Corynebacterium*, and *Bifidobacterium* emerging as key biomarkers, making them important targets for interventions to promote long-term respiratory health in preterm infants.
- To recognize microbial predictors of recurrent wheezing in preterm infants could allow to explore potential microbiota-modulating strategies to mitigate respiratory complications in this high-risk population

## Introduction

The human microbiota plays a fundamental role in modulating the immune system and influencing the development of respiratory and gastrointestinal diseases.^1^ The nasopharyngeal microbiota serves as a first line of defence against inhaled pathogens,^2^ while the intestinal microbiota shapes immune maturation and systemic inflammatory responses.^3,4^ These microbial communities interact with the host from birth, contributing to long-term immune programming.^5^

Premature neonates exhibit significantly altered microbiota due to immunological immaturity, frequent antibiotic use, and formula feeding instead of breast milk.^6,7^ This dysbiosis is associated with an increased risk of respiratory infections and inflammatory conditions.^8,9^ Additionally, the structural immaturity of the lungs and gut exacerbates their immune vulnerability.^10,11^

The gut-lung axis exemplifies the bidirectional interaction between the respiratory and gastrointestinal systems, mediated by microbial metabolites, cytokines, and immune cells migrating between organs.^12,13^ Recent studies indicate that intestinal dysbiosis can influence susceptibility to pulmonary diseases, including asthma and wheezing.^14,15^ In preterm infants, this relationship is critical given their heightened risk for respiratory infections and bronchopulmonary dysplasia.^16,17^ Early viral infections, particularly with respiratory syncytial virus (RSV), have been linked to allergic sensitization and subsequent asthma development.^18,19^ Nasopharyngeal microbiota influences the severity of these infections, affecting the risk of recurrent wheezing.^20,21^ Children with microbiota profiles dominated by *Moraxella catarrhalis* and *Haemophilus influenzae* are at higher risk of severe respiratory episodes.^22,23^ In preterm infants, these pathogens are particularly prevalent, increasing the likelihood of chronic lung disease.^24,25^

Longitudinal studies focusing on respiratory and gastrointestinal samples in preterm infants provide valuable insights into these interactions.^26,27^ Real-time PCR-based detection of respiratory viruses and microbiota profiling has become a key tool for investigating the role of microbial dynamics in preterm health outcomes.^28^ These approaches enable the identification of microbial biomarkers associated with recurrent wheezing, providing a basis for potential therapeutic interventions.^29^ Multiple studies highlight that the initial microbial composition influences wheezing prevalence in preterm children.^30^ Reduced bacterial diversity and the presence of pathogens such as *Staphylococcus aureus* correlate with higher respiratory disease incidence.^31,32^ Conversely, colonization with *Lactobacillus* and *Bifidobacterium* is associated with a more benign inflammatory profile.^33,34^ Our previous findings demonstrate a significant relationship between early nasopharyngeal microbiota composition and the incidence of recurrent wheezing in preterm infants.^35^ A higher abundance of respiratory pathogens correlates with increased hospitalizations due to viral infections.^36,37^

In this multicenter prospective study conducted in two neonatal intensive care units (NICUs) in Madrid, Spain, we analyzed the association between nasopharyngeal and gut microbiota profiles, viral infections, and the development of recurrent wheezing in preterm infants. By integrating epidemiological, clinical, and microbiological data, this study aims to identify microbial and viral predictors of recurrent wheezing and to explore potential microbiota-modulating strategies to mitigate respiratory complications in this high-risk population.^35,38^

## Methods

### Study design

A prospective multicentre study (2019–2022) at two Madrid NICUs (H. Severo Ochoa and H. La Paz), investigated respiratory viral infections and microbiota profiles in preterm infants born at <32 weeks’ gestational age and <8 days old at the time of inclusion. Infants were excluded if they had major malformations, chromosomal disorders, immunodeficiencies, or if their parents refused participation. Ethical approval was obtained (ref. PI4676) and written informed consent was signed by the parents or legal guardians.

### Outcome variables and definitions

The main clinical outcome was wheezing, defined as ≥2 episodes of physician-diagnosed wheezing requiring medical attention during the first year of life. Additional outcomes included respiratory admissions, anti-asthmatic treatment prescription, and atopy (defined as dermatitis and/or food allergy).

### Biological sample collection

All samples were collected during the initial hospitalization for prematurity. Two simultaneous NPAs samples were collected during the first week of life (specifically between 3 and 7 days of life). One of them was kept refrigerated at 4 °C, until its transport to the National Centre for Microbiology (ISCIII) for virological study, and the other one, was used for the microbiota study and stored immediately at -80°C until use. Additionally, weekly samples of NPA were collected for virological study.

Faecal samples were also collected between days 3 and 7 of life, following a standardized protocol and using sterile techniques. Samples were immediately stored at -80°C until analyses. Sample collection was performed by trained neonatal nurses.

### Determination of virus on nasopharyngeal aspirates (NPA)

RNA and DNA from 200 µL aliquots of NPA were extracted using the QIAamp MinElute Virus Spin Kit in an automated extractor (QIAcube, Qiagen, Valencia, Spain) from the NPA samples stored for the National Centre for Microbiology (ISCIII) for virological study. Respiratory virus detection was performed by four independent real-time multiplex PCR (RT-PCR) assays using the SuperScript III Platinum One-Step Quantitative RT-PCR System (Invitrogen®, Waltham, MA). The first assay detected Influenza A, B, and C viruses; the second assay detected parainfluenza viruses 1 to 4 (PIV), hRV, and enteroviruses; the third assay detected RSV types A and B, human metapneumovirus (hMPV), human bocavirus (hBoV), and AdV. Human coronavirus (HCoV) was investigated using a generic RT-PCR that was able to detect human alpha and beta coronaviruses, HCoV 229E/HCoV NL63, and HCoV OC43/HCoV HKU1, respectively. The primers and Taqman probes used in the study had already been reported by the study investigators^.^^39^ In addition, detection of SARS-CoV-2 was performed on an extracted RNA from NPAs from 2020 using a real-time RT-PCR assay based on the method designed by Corman et al.,^40^ for the specific amplification of the E gene using the One-Step RT-PCR Kit (NZYTech, Lisbon, Portugal).

### Microbiota profiling

#### DNA extraction and 16S rRNA amplicon sequencing

Total DNA was extracted from paired NPAs (500ul) and faecal material (approx. 100 mg) using the automated assisted method based on magnetic beads (Maxwell® RSC Instrument coupled with Maxwell RSC Pure Food GMO and authentication kit, Promega, Spain) following the manufacturer’s instructions with previous treatments to improve the DNA extraction. In brief, samples were treated with lysozyme (20 mg/mL) and mutanolysin (5 U/mL) for 60 min at 37 °C and a preliminary step of cell disruption with 3-μm diameter glass beads during 1 min at 6 m/s by a bead beater FastPrep 24-5 G Homogenizer (MP Biomedicals). Purification of the DNA was performed using DNA Purification Kit (Macherey-Nagel, Duren, Germany) according to manufacturer’s instructions and DNA concentration was measured using Qubit® 2.0 Fluorometer (Life Technology, Carlsbad, CA) for further analysis.

Amplicon libraries of the 16S rRNA gene were generated with a protocol from Illumina (V3-V4 variable region), were treated as described in the Illumina protocol, and in Cabrera-Rubio R et al.^27^ Samples were sequenced on Illumina paired-end (2×250 bp) on a NovaSeq- PE250 Illumina platform (Novogene Bioinformatics Technology Co., Ltd) according to manufacturer instructions. Controls during DNA extraction and PCR amplification were also included and sequenced.

#### Bioinformatic and statistical analyses

16S rRNA gene sequencing data were processed with a modified pipeline described by Cabrera-Rubio et al.^27^ Sequencing data were quality-filtered (removal of low- quality nucleotides at the 3’ end in windows of 20 nt, sequences with less than 200nt, adapter removal and deduplication) with fastp programme.^41^ After that, sequences were joined with VSEARCH,^42^ and were denoised with Deblur,^43^ with default parameters. Amplicon Sequence Variants (ASVs) mapping to the human genome (GRCh38) using the Burrow- Wheeler Aligner in Deconseq v0.4.3 were filtered out.^44^ The R package Decontam was applied to control for potential contaminants.^45^ All samples with less than 5000 reads were eliminated for the analysis. ASVs were annotated with a Naïve-Bayes classifier based on the scikit-learn system and the RDP database.^46^ The ASVs were aligned with MAFFT,^47^ to then make a phylogenetic tree with FASTTREE,^48^ that was then midpoint-rooted.

#### Consideration of centre effects

Since samples were obtained from two different NICUs (HSO and HULP), potential centre-specific variability was taken into account in all statistical analyses. Centre was included as a stratification or grouping factor in the alpha and beta diversity analyses, differential abundance testing, and clinical outcome comparisons (e.g., wheezing). When appropriate, analyses were also performed separately within each hospital to confirm the robustness of the results. Differential abundance analyses didn’t reveal distinct microbial signatures by hospital (with statistical significance). This approach allowed us to control for potential intra-hospital dependencies and hospital-related confounding, as recommended in previous multi-centre microbiome studies.^49^

#### Prediction models

Binary clinical variables such as sex and delivery mode were encoded as numeric values (0/1) prior to modelling, in order to facilitate integration with continuous microbiome predictors in the random forest algorithm, which was used to predict the binary outcomes of interest. While this numeric encoding can partially reduce the known variable importance bias in standard random forests, it does not fully eliminate it, especially when compared to variables with multiple split points or continuous distributions.^50^

To address this, we additionally implemented conditional inference forests using the cforest function from the party package in R, following the unbiased recursive partitioning framework described by Hothorn et al. 2006.^51^ This method reduces selection bias by separating variable selection and splitting criteria and is more appropriate when working with mixed-type predictors. Variable importance was calculated using conditional permutation (varimp with conditional = TRUE), and both sets of results (standard and conditional random forest) were compared. In both cases, the random forest models were applied to evaluate the predictive contribution of clinical and microbiome variables. The conditional model confirmed the robustness of the microbial predictors identified in the original analysis, while providing a less biased estimate of the relative importance of clinical covariates.

In parallel, to further characterize predictive microbial signatures, we applied the microbial balance approach using the selbal.cv() function from the selbal R package.^29^ This function implements repeated k-fold cross-validation to identify robust balances of microbial taxa associated with a binary outcome. We used 5-fold cross-validation repeated 10 times (n.fold = 5, n.iter = 10) to ensure robustness. At each iteration, a logistic regression model was trained on the training folds, and the area under the receiver operating characteristic curve (AUC) was computed on the held-out fold to assess out-of-sample predictive performance.^52^ The optimal microbial balance was selected based on the highest average AUC across all iterations.

#### Statistical analysis

Since samples were obtained from two different NICUs (HSO and HULP), potential centre-specific variability was taken into account in all statistical analyses. Centre was included as a stratification or grouping factor in the alpha and beta diversity analyses, differential abundance testing, and clinical outcome comparisons (e.g., wheezing). When appropriate, analyses were also performed separately within each hospital to confirm the robustness of the results. This approach allowed us to control for potential intra-hospital dependencies and hospital-related confounding, as recommended in previous multi-centre microbiome studies.^53^

Multivariate analyses assessing the relationship between microbiota features and wheezing were adjusted for key clinical covariates, including gestational age (continuous), presence of BPD (yes/no), and antibiotic exposure (categorized by timing and duration). These variables were selected based on prior knowledge of their potential influence on both microbiota development and respiratory outcomes.

Alpha-diversity indices (Chao1, Simpson and Shannon, and differences with Anova tests) and beta diversity (Bray-Curtis and Unifrac, with PERMANOVA with vegan R package^54^ were obtained using phyloseq^55^ R package. Rarefied was not performed, but a filter of 0.01% relative abundance in a minimum of 25% of the samples was established to perform the following analyses. To determine the most appropriate statistical method, we first applied the Shapiro-Wilk test^56^ to assess whether the data followed a normal distribution. The data were not normally distributed. Since the results indicated that the data were non-parametric, we employed Spearman’s rank correlation coefficient to evaluate the association between variables. Spearman correlations were calculated using Phyloseq^55^ in R programme version 4.1.2.^57^ Pairwise Spearman’s rank correlations were performed to assess potential ecological relationships between bacterial genera and to explore associations with clinical variables. Given the non-normal and zero-inflated distribution of microbiota data, even after log transformation, Spearman’s correlation was selected over parametric alternatives. Binary clinical variables (e.g., delivery mode, antibiotic use) were numerically coded (0/1) for inclusion in the analyses. Locally Estimated Scatterplot Smoothing curves were applied to visualize monotonic trends without assuming linearity. This non-parametric approach is widely used in exploratory microbiome analyses, particularly when integrating binary and continuous predictors, and serves as an efficient first-pass screening tool to complement downstream multivariate methods (e.g., RDA, LEfSe, and Random Forest). These analyses were exploratory, unadjusted for covariates or multiple testing, and should therefore be interpreted with caution. All correlation analyses and visualizations were conducted using the ggpubr R package version 0.6.0.^58^ Scatterplots included in correlation analyses display trend lines for visual guidance only, and LOESS smoothing curves with 95% confidence intervals were applied to better illustrate monotonic trends. Spearman’s rank correlation coefficients are reported, as linearity was not assumed. Associations between microbiota and wheezing were analyzed via balanced selection.^29^ In addition, a linear discriminant analysis effect size (LefSe); Segata et al.^59^ was performed to discover specific bacterial biomarkers. All P values were adjusted using False discovery rate (FDR), based on the Benjamini and Hochberg (BH) method.

In addition to univariate comparisons, multivariate logistic regression models were used to evaluate the association between individual genera and wheezing development, adjusting for relevant clinical covariates including gestational age, sex, delivery mode, antibiotic use, and respiratory support. These models allowed us to account for potential confounding and assess the independent predictive value of microbial taxa. Odds ratios (OR) and 95% confidence intervals (CI) were calculated to aid interpretation.

## Results

### Patients and clinical data

During the study period, 91 preterm newborns were included, of which 48 (52.7%) were female (Table 1). One patient died at 21 days of life; all the remaining survived. Up to 22 infants (24.1%) were born at less than 28 weeks of gestational age (GA), 72 (78.3%) were born by caesarean section, and most of them received breast milk (n = 84, 91.3%). Antibiotic treatment was prescribed in 49 (53.8%) patients, but only 41 received them before the sample was taken: of them 30 infants received antibiotics for early-onset sepsis (median duration: 4 days, IQR 3–7), and 11 for late-onset sepsis (median: 6 days, IQR 5–10). The median gestational age was 30 weeks with an interquartile range (IQR) of 28-31. All the remaining survived, and 25 (27.2%) developed bronchopulmonary dysplasia (BPD). During their stay in the NICU, a respiratory virus infection was detected in 19 infants (20.9%) rhinovirus being the most frequent.

**Table 1:** Clinical data of the preterm infants

	*N* = 91 infants
**Admission in NICU**	
Sex (male)	43 (46.7%)
Gestational age	
≤ 28 weeks	22 (24.1%)
>28 weeks	69 (75.8%)
Type of delivery	
Caesarean section	72 (78.3%)
Vaginal birth	20 (21.7%)
Probiotics	12 (13.0%)
Antibiotics	49 (53.8%)
Before sample taken	41 (45.0%)
Risk of infection/ early-onset sepsis	30
Late-onset sepsis	11
Mechanical ventilation (MV)	84 (92.3%)
Days of MV: median (IQR)	4 (2–12)
BDP	25 (27.2%)
Viral infection	19 (20.9%)
Rhinovirus	8/19
Coronavirus	3/19
Adenovirus	3/19
RSV	1/19
Bocavirus	1/19
Coinfections	3/19
**Follow up**	
Breastfed	83 (91.2%)
Median months of breastfed (IQR)	6 (2.75–10)
Wheezing episodes	22 (23.9%)
Less than 2 episodes	15 (16.3%)
More than 3 episodes	7 (7.7%)
Respiratory admissions	12 (13.2%)
Anti-asthmatic treatment	10 (10.9%)
Atopy (dermatitis or food allergy)	28 (30.4%)

*NICU* neonatal intensive care unit, *BPD* bronchopulmonary dysplasia, *IQR* interquartile range, *MV* mechanical ventilation, *RSV* respiratory syncytial virus.

In 63 patients, both a stool sample and an NPAs were collected in the first week of life (between 3-8 days), with 17 patients having only stool samples and with 11 having only NPAs, bringing the total to 91 neonates. This group included infants both with and without a diagnosis of BPD. At follow-up, at one year of age, 22 (24.4%) of children had developed wheezing (≥2 physician-confirmed episodes), and 7 (7.7%) had suffered more than 3 episodes. Of them, 12 (13.2%) required admission and 10 (11.2%) needed anti-asthmatic chronic treatment (leukotrienes antagonists or inhaled corticosteroids). The main clinical data are shown in Table 1.

Table 2 summarizes the clinical comparisons between wheezing and non-wheezing infants. Caesarean delivery was less frequent among wheezing infants (59.1% vs. 83.8%, p = 0.036), and other factors such as sex, gestational age, and mechanical ventilation did not differ significantly.

**Table 2:** Comparison of risk factors during the neonatal period between children with or without development or recurrent wheezing

	Wheezing *N* = 22/90	No wheezing *N* = 68/90	*p*; OR (IC 95%)
Sex (male)	7 (31.8%)	35 (51.5%)	*p* = 0.142; 0.533 (0.241–1.182)
Gestational age ≤ 28 weeks	5 (22.7%)	17 (25.0%)	*p* = 0.538; 0.909 (0.380–2.178)
Type of delivery			
Caesarean section	13 (59.1%)	57 (83.8%)	***p*** = **0.036; 0.279** (**0.096–0.810**)
Breastfeeding	20 (90.9%)	63 (92.6%)	*p* = 0.548; 1.186 (0.346–4.065)
Probiotics	4 (18.2%)	8 (11.8%)	*p* = 0.478; 1.444 (0.589–3.542)
Antibiotics	10 (45.5%)	38 (55.9%)	*p* = 0.465; 0.729 (0.351–1.513)
Mechanical ventilation	21 (95.2%)	62 (91.2%)	*p* = 0.452; 1.771 (0.278–11.285)
Virus during NICU	3 (13.6%)	15 (22.1%)	*p* = 0.544; 0.632 (0.210–1.902)
BDP	5 (22.7%)	19 (27.9%)	*p* = 0.874; 0.809 (0.335–1.952)

*NICU* neonatal intensive care unit, *BDP* bronchopulmonary dysplasia.

### Which factors contribute the most to the preterm microbiota?

For this study, we first assessed the association between baseline clinical variables (gestational age, delivery mode, antibiotics, BPD, etc.) and microbiota diversity and structure. These variables were then included in downstream multivariate models evaluating their contribution to wheezing development.

In the NPA samples, significant differences in alpha diversity were observed for the following factors (Figure not shown): **Gestational Age;** Higher richness was observed in samples from infants with a gestational age greater than 32 weeks (Chao1 - *P*-value < 0.05; Observed - *P*-value < 0.05), **Probiotics;** Greater diversity was identified in the group receiving probiotics (Chao1 - *P*-value < 0.05; Observed - *P*-value < 0.05), **Sex**: Higher diversity was found in males compared to females (Shannon - *P*-value < 0.05; Simpson - *P*-value < 0.05) and **Lactation;** In non-wheezing samples, formula-fed infants exhibited higher richness (Chao1 - *P*-value < 0.05; Observed - *P*-value < 0.05), a difference that was not observed in samples from infants who developed wheezing.

The Bray-Curtis distance matrix analysis revealed a significant impact of delivery mode (R^2^ = 0.0392, *P* = 0.009), Gestational age (R^2^ = 0.021, *P* = 0.049), Sex (R^2^ = 0.0392, *P* = 0.022), antibiotic-treatment in ICU (R^2^ = 0.025, *P* = 0.02), and wheezing (R^2^ = 0.0392, *P* = 0.031). Similarly, the Unweight Unifrac distance matrix showed a significant association with gestational age (R^2^ = 0.054, *P* = 0.003), Sex (R^2^ = 0.029, *P* = 0.028) and wheezing (R^2^ = 0.0392, *P* = 0.031).

Regarding intestinal samples, significant differences in alpha diversity were identified for the following factors (data not shown): Gestational Age; Infants with a gestational age greater than 32 weeks exhibited higher richness (*Chao1* - *P*-value < 0.05; *Observed* - *P*-value < 0.05), Bronchopulmonary Dysplasia (BPD); Higher richness and diversity were observed in the non-BPD group (*Chao1* - *P*-value < 0.05; *Observed* - *P*-value < 0.05; *Shannon* - *P*-value < 0.05; *Simpson* - *P*-value < 0.05)

Bray-Curtis distance matrix showed a relevant impact on delivery (R^2^ = 0.029, *P* = 0.009), Sex (R^2^ = 0.022, *P* = 0.042) and antibiotic-treatment in ICU (R^2^ = 0.024, *P* = 0.02). With Unifrac (Unweight) distance matrix showed a relevant impact on Gestational age (R^2^ = 0.054, *P* = 0.003), Sex (R^2^ = 0.029, *P* = 0.028) and wheezing (R^2^ = 0.045, *P* = 0.047). Table 3 summarizes the factors associated with beta diversity based on the Bray-Curtis and Unweighted Unifrac distance matrices. It presents the R² and P values for factors, intestinal samples (Gut), and NPA samples, highlighting the significant associations identified in the analysis.

**Table 3:** Summarizes the factors associated with beta diversity based on the Bray-Curtis and Unweighted Unifrac distance matrices.

	Distance matrix	Delivery		Antibiotic-treatment		Gestational age		Wheezing	
**Gut**	Bray-Curtis	R2 = 0.029	*P* = 0.009	R2 = 0.024	*P* = 0.02	-	-	-	-
	Unifrac (Unweight)	-	-	-	-	R2 = 0.054	*P* = 0.003	R2 = 0.045	*P* = 0.047
**NPA**	Bray-Curtis	R2 = 0.0392	*P* = 0.009	R2 = 0.025	*P* = 0.02	R2 = 0.021	*P* = 0.049	R2 = 0.0392	*P* = 0.031
	Unifrac (Unweight)	-	-	-	-	R2 = 0.054	*P* = 0.003	R2 = 0.0392	*P* = 0.031

*NPA* nasopharyngeal aspirate.

Our analysis revealed that the composition and diversity of the microbiota in both NPA and intestinal samples of preterm neonates were significantly influenced by clinical factors. These factors (such as delivery mode, antibiotic exposure, and type of feeding) are known to shape microbial communities early in life and may contribute to respiratory outcomes such as wheezing. Specifically, caesarean delivery was associated with a higher prevalence of genera such as *Klebsiella* and *Enterococcus*, while antibiotic use reduced overall microbial diversity and suppressed beneficial genera like *Bifidobacterium*. Breastfeeding, on the other hand, supported the growth of immunomodulatory bacteria due to its high content of oligosaccharides.

## Microbiota composition and clinical associations

### General characterization of NPA microbiota and biomarkers

**Fig. 1:**
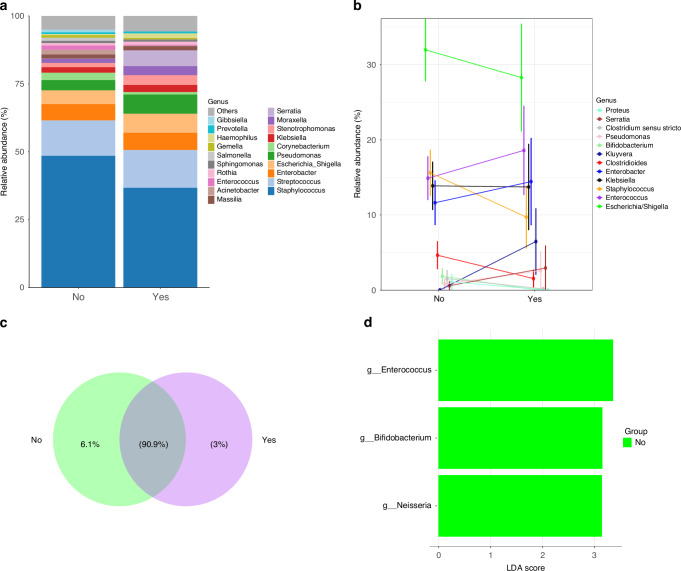
Taxonomy, core of ASVs, and Statistical significances with LEfSe from nasopharyngeal aspirate (NPA) in the patients with wheezing vs the non-wheezing group. **a**, **b** Relative abundance of ASVs according to wheezing in NPA and **c** shared by all the study groups. **d** Linear discriminant analysis (LDA) effect size (LefSe) in NPA samples.

The most abundant genera in NPA samples were *Staphylococcus*, *Streptococcus*, *Enterobacter*, *Escherichia/Shigella*, *Pseudomonas*, *Corynebacterium*, *Klebsiella*, *Stenotrophomonas*, *Moraxella*, and *Serratia*, with highly variable percentages depending on the development of wheezing, as shown in Fig. 1a. Similar to the previous samples, the percentages varied significantly depending on the presence or absence of recurrent wheezing during follow-up (Fig. 1b). Regarding the shared ASVs between the wheezing and non-wheezing groups, Fig. 1c shows that the core of ASVs between both groups is 90.9%, exclusive to the non-wheezing group is 6.1% and for the wheezing group is 3%. Statistical significance of their differential distribution according to the wheezing groups was assessed with LEfSe. NPAs samples showed significant differences were found in the non-wheezing group with *Enterococcus, Bifidobacterium* and *Neisseria* (Fig. 1d).

**Fig. 2:**
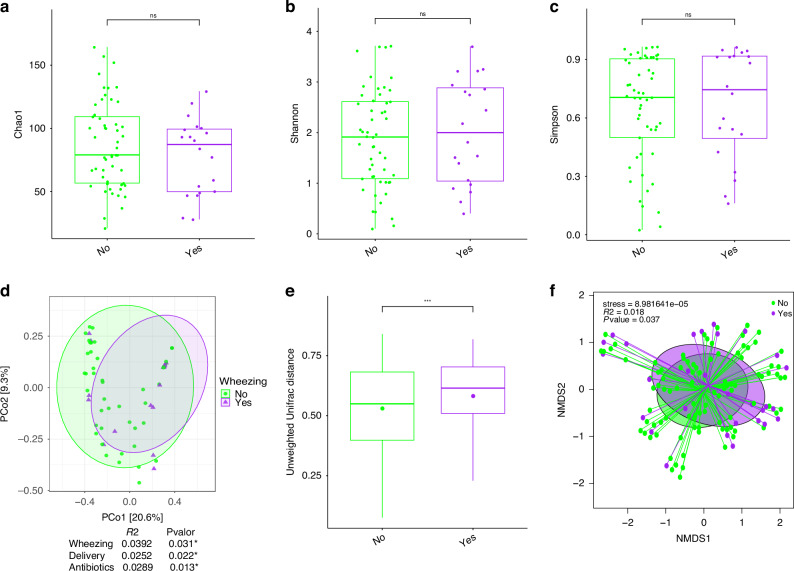
Alpha and beta diversity from nasopharyngeal aspirate (NPA) microbiota in the patients with wheezing vs non-wheezing. **a**–**c** Boxplot showing richness, Shannon and Simpson diversity indices according to the wheezing and non-wheezing groups in the NPA samples. **d** Principal coordinates analysis (PCoA) plot based on Bray-Curtis dissimilarities of nasopharyngeal (**e**) and Bray-Curtis distance microbiota composition of samples from all patients included in the study. **f** Non-metric multidimensional escalation (NMDS) plot based on Bray-Curtis dissimilarities of nasopharyngeal. *P*-value corresponds to the Adonis PERMANOVA test.

Alpha-diversity metrics did not show significant differences in NPA microbiota composition concerning wheezing development (Fig. 2a–c). However, beta-diversity analyses using the Bray-Curtis distance matrix revealed a significant impact of wheezing on NPA microbiota composition (Fig. 2d–f). Specifically, the microbiota was significantly associated with wheezing episodes (*R²* = 0.0392*, P* = 0.031). The “envfit” function is a very powerful tool in ecology, microbiology and other related fields, as it allows to identify and quantify the influence of environmental factors on the beta diversity of a biological community. To further explore the relationships between microbiota composition and clinical variables in relation with wheezing, the Bray-Curtis distance matrix was analyzed with the “envfit” function. This analysis identified significant differences based on the mode of delivery (*R²* = 0.0256*, P* = 0.022) and prior antibiotic use (*R²* = 0.0289*, P* = 0.013) (Fig. 1d).

**Fig. 3:**
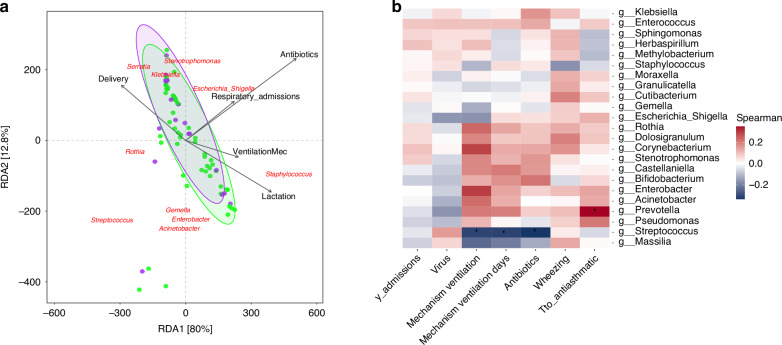
Statistically significant taxonomic change in wheezing and non-wheezing samples in nasopharyngeal aspirate (NPA). **a** Triplot of RDA showing the distribution of NPA samples with reference to bacterial genera and explanatory variables. The ellipses are drawn containing 75% of each group of samples from each study group, and coloured accordingly. The arrows indicate the direction and strength (length) of the explanatory variables. The red names correspond to the higher abundance of each bacterial genus. **b** Spearman correlation analysis.between binary-coded clinical factors and genus-level microbial abundances.

The behaviour of significant variables influencing beta diversity was further explored using RDA with the Bray-Curtis matrix (Fig. 3a), a multivariate analysis was done. In NPA samples, antibiotic use (*R²* = 0.159*; P* = 0.003) and mode of delivery (*R²* = 0.0794*; P* = 0.049) were the most relevant factors. Wheezing was associated with the genera *Serratia*, *Klebsiella*, and *Stenotrophomonas*, which were aligned with respiratory admissions and antibiotic use, and these factors were aligned with the genera *Escherichia/Shigella*. By contrast, *Staphylococcus* was associated with breastfeeding and non-wheezing samples. Separated along the second coordinate (RDA2), mechanical ventilation and breastfeeding were both associated with the genus *Staphylococcus* and were predominantly linked to non-wheezing samples in the RDA plot. Furthermore, in a different quadrant along the same coordinate, the genera *Gemella*, *Enterobacter*, and *Streptococcus* were similarly associated with samples from non-wheezing children.

To evaluate the relationship between various factors and the genera, a Spearman correlation analysis was conducted. As shown in Fig. 3b, significant negative correlations were identified between the genus *Streptococcus* and the factors mechanical ventilation, duration of mechanical ventilation, and antibiotic prescription.

By integrating the findings from the RDA and Spearman’s correlation analyses, a weak positive correlation (coefficient of 0.18 (*P* = 0.044)) was observed between *Moraxella* and *Corynebacterium* in nasopharyngeal samples (Fig. S1a), consistent with previous reports of co-occurrence during early colonization. However, these relationships were not associated with wheezing status. Additional analyses revealed a strong positive correlation between *Dolosigranulum* and *Moraxella* (coefficient of 0.38, *P* = 1.6 × 10⁻⁵; Fig. S1B) as well as between *Bifidobacterium* and *Enterococcus* (coefficient of 0.47, *P* = 5.8 × 10⁻⁸; Fig. S1c). *Dolosigranulum* also showed strong positive associations with *Enterococcus* (coefficient of 0.38, *P* = 1.4 × 10⁻⁵; Fig. S1d) and with *Corynebacterium* (coefficient of 0.32, *P* = 3.3 × 10⁻⁴; Fig. S1e). None of these associations were significantly linked to wheezing status, suggesting that these microbial co-occurrences may represent stable ecological relationships independent of early respiratory symptoms. Similar to NPAs, alpha-diversity analyses in gut microbiota showed no significant differences between wheezing and non-wheezing groups (Fig. 5a–c). However, beta-diversity analysis with the Bray-Curtis distance matrix showed a significant association between gut microbiota composition and wheezing episodes (R^2^ = 0.0392, *P* = 0.031) (Fig. 5d–f). With the intestinal samples (Fig. 5d), the “envfit” function in relation with wheezing factor found as significant factors mechanic ventilation during NICU admission (R^2^ = 0.224; *P* = 0.001), with antibiotics use (R^2^ = 0.329; *P* = 0.001) and lactation (R^2^ = 0.265; *P* = 0.001), We also found respiratory admissions close to significance (R^2^ = 0.052; *P* = 0.097).

Finally, the multivariate logistic regression models had several bacterial genera showed statistically significant associations with the studied condition after adjustment for multiple comparisons. Among those with inverse associations, *Bifidobacterium* (OR = 0.968; 95% CI: [0.93, 0.99]; adjusted p = 0.0004) and *Enterococcus* (OR = 0.977; 95% CI: [0.94, 1.00]; adjusted *p* = 0.0012) exhibited the strongest effects, suggesting a reduced relative abundance in individuals exposed to the condition. *Vagococcus* also displayed a markedly decreased association (OR = 0.182; 95% CI: [0.01, 0.82]; adjusted *p* = 0.0042), potentially indicating a substantial depletion in this taxon. Additionally, other genera such as *Neisseria*, *Sphingomonas*, *Streptococcus*, and *Staphylococcus* demonstrated statistically significant inverse associations, albeit with odds ratios close to 1, suggesting subtle but consistent shifts in microbial abundance. Conversely, *Serratia* was the only genus positively associated with the condition, showing a modest but statistically significant increase in relative abundance (OR = 1.047; 95% CI: [1.01, 1.10]; adjusted *p* = 0.0002). Other genera, including *Fusobacterium*, *Citrobacter*, *Dolosigranulum*, *Corynebacterium*, *Escherichia/Shigella*, and *Klebsiella*, had odds ratios close to unity with marginal statistical significance, which may reflect minor associations or limited statistical power. Overall, these findings suggest that specific shifts in the microbial composition are associated with the condition under study, with some taxa potentially playing protective or pathogenic roles depending on the direction and magnitude of association.

### General characterization of stool microbiota and biomarkers

**Fig. 4:**
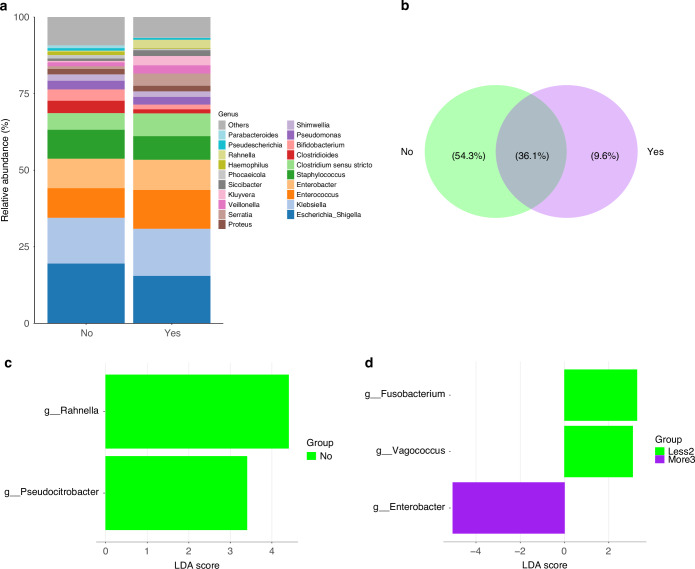
Taxonomy, core of ASVs and Statistical significances with LEfSe from gut samples in the patients with wheezing vs non-wheezing group. **a** Relative abundance of ASVs according to wheezing in NPA and **b** shared by all the study groups. **c**, **d** Linear discriminant analysis (LDA) effect size (LefSe) in NPA samples with wheezing vs non-wheezing (**c**) and number of wheezing episodes in the first 6 months of life.

The most abundant genera in gut samples corresponded to *Escherichia/Shigella, Enterococcus, Staphylococcus, Klebsiella, Enterobacter, Clostridioides, Kluyvera, Bifidobacterium, Pseudomonas, Clostridium sensu stricto* and *Serratia*. As in NPA samples, relative abundances varied significantly depending on wheezing development (Fig. 4a). In gut samples, the proportion of shared ASVs between wheezing and non-wheezing groups was 36.1%, with 54.3% unique to the non-wheezing group and 9.6% unique to the wheezing group (Fig. 4b). LEfSe analysis identified significant genera in the non-wheezing group, including *Klebsiella*, *Bifidobacterium*, and *Kosakonia* (Fig. 4c). When comparing gestational age groups (<28 weeks vs. ≥28 weeks), specific genera such as *Fusobacterium* and *Vagococcus* were more abundant in the <28-week group (Fig. 4d). Additionally, children with more than three wheezing episodes were significantly associated with the genus *Enterobacter*.

**Fig. 5:**
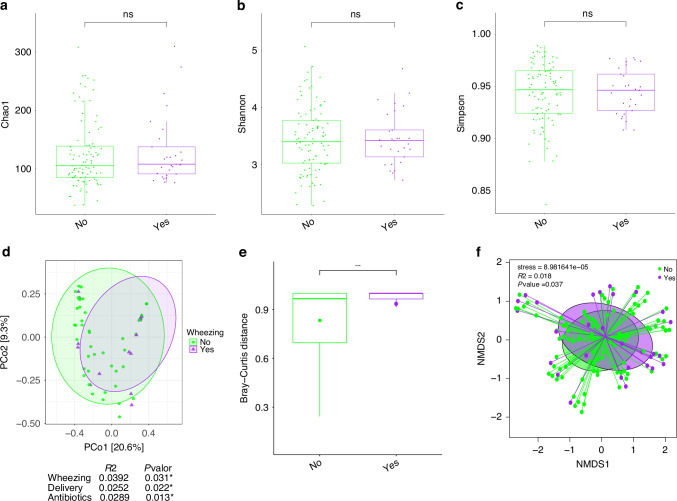
Alpha and beta diversity from gut microbiota in the patients with wheezing vs non-wheezing. **a**–**c** Boxplot showing richness, Shannon and Simpson diversity indices according to the wheezing and non-wheezing groups in gut samples. **d** Principal coordinates analysis (PCoA) plot based on Bray-Curtis dissimilarities of gut (**e**) and Bray-Curtis distance microbiota composition of samples from all patients included in the study. **f** Non-metric multidimensional escalation (NMDS) plot based on Bray-Curtis dissimilarities of gut samples. *P*-value corresponds to the Adonis PERMANOVA test.

Similar to NPAs, alpha-diversity analyses in gut microbiota showed no significant differences between wheezing and non-wheezing groups (Fig. 5a–c). However, beta-diversity analysis with the Bray-Curtis distance matrix showed a significant association between gut microbiota composition and wheezing episodes (R^2^ = 0.0392, *P* = 0.031) (Fig. 5d–f). With the intestinal samples (Fig. 5d), the “envfit” function in relation with wheezing factor found as significant factors mechanic ventilation during NICU admission (R^2^ = 0,224; P = 0.001), with antibiotics use (R^2^ = 0,329; *P* = 0.001) and lactation (R^2^ = 0,265; *P* = 0.001), We also found respiratory admissions close to significance (R^2^ = 0,052; P = 0.097).

**Fig. 6:**
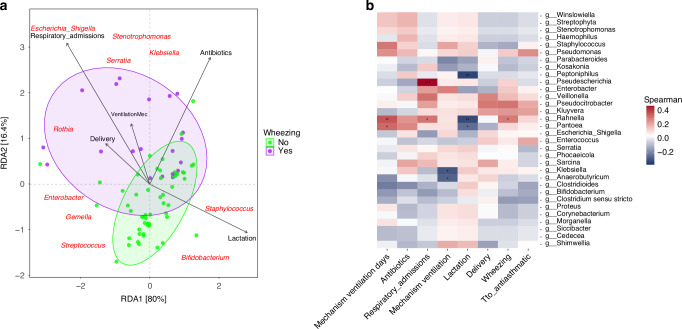
Statistically significant taxonomic change in wheezing and non-wheezing samples in gut samples. **a** Triplot of RDA showing the distribution of gut samples with reference to bacterial genera and explanatory variables. The ellipses are drawn containing 75% of each group of samples from each study group and coloured accordingly. The arrows indicate the direction and strength (length) of the explanatory variables. The red names correspond to the higher abundance of each bacterial genus. **b** Spearman correlation analysis factors against genus level.

To evaluate the influence of significant variables on beta diversity, we projected the data onto an RDA plot using a Bray-Curtis distance matrix, a multivariate analysis in intestinal samples (Fig. 6a), the “envfit” function identified mechanical ventilation during NICU admission (R² = 0.224, *P* = 0.001), antibiotic use (R² = 0.329, P = 0.001), and lactation (R² = 0.265, *P* = 0.001) as significant factors. Respiratory admissions were also noted to approach significance (R² = 0.052, *P* = 0.097). The development of wheezing was associated with variables such as respiratory admissions, mode of delivery, and mechanical ventilation during NICU admission, as well as with specific genera, including *Escherichia/Shigella*, *Serratia*, *Stenotrophomonas*, and *Rothia*. Infants with wheezing development were separated along the first coordinate (RDA1) and were strongly associated with antibiotic use and the genus *Klebsiella*. Conversely, infants who did not develop wheezing were linked to breastfeeding, which was closely associated with the genera *Bifidobacterium* and *Staphylococcus*. These findings highlight distinct microbial and clinical profiles linked to the development or absence of wheezing.

In the infant gut microbiota, Spearman correlation analysis (Fig. 6b) revealed several notable associations. The genus *Rahnella* exhibited positive correlations with wheezing, respiratory admissions, and duration of mechanical ventilation, as well as with breastfeeding. Similarly, the genus *Pantoea* showed a positive correlation with breastfeeding and a negative correlation with the duration of mechanical ventilation. Additionally, *Klebsiella* and *Anaerobutyricum* demonstrated positive correlations with mechanical ventilation. These findings underscore the complex interactions between clinical factors and gut microbial composition in infants.

In the multivariate logistic regression models analysis, none of the evaluated bacterial genera exhibited statistically significant associations with the condition after adjustment for multiple comparisons. The genera *Rahnella* (OR = 1.001; 95% CI: [0.97, 1]; adjusted *p* = 0.0431) and *Pseudocitrobacter* (OR = 1.006; 95% CI: [0.97, 1]; adjusted *p* = 0.0489) were the only taxa with adjusted p-values below 0.05. However, their odds ratios were very close to 1, and their confidence intervals included or approached the null value, suggesting negligible or uncertain biological relevance. Several genera presented extreme or highly imprecise odds ratio estimates, such as *Fusobacterium* (OR = 55.18; 95% CI: [0, NA]) and *Rothia* (OR = 0.00032; 95% CI: [0, 4.34e + 57]), reflecting a high degree of variability and lack of statistical power. These values are not interpretable as meaningful associations due to the wide, undefined confidence intervals and very high adjusted *p*-values (*p* > 0.99 in both cases). Most other genera, including *Klebsiella*, *Staphylococcus*, *Enterococcus*, *Serratia*, *Pseudomonas*, and *Clostridium sensu stricto*, exhibited odds ratios very close to 1 with tight or fixed confidence intervals, and none showed statistical significance (adjusted *p*-values > 0.1). These results suggest the absence of strong or consistent microbial shifts in relation to the condition under study.

## Prediction of recurrent wheezing development by integrating microbial and perinatal factors using Random Forest

**Fig. 7:**
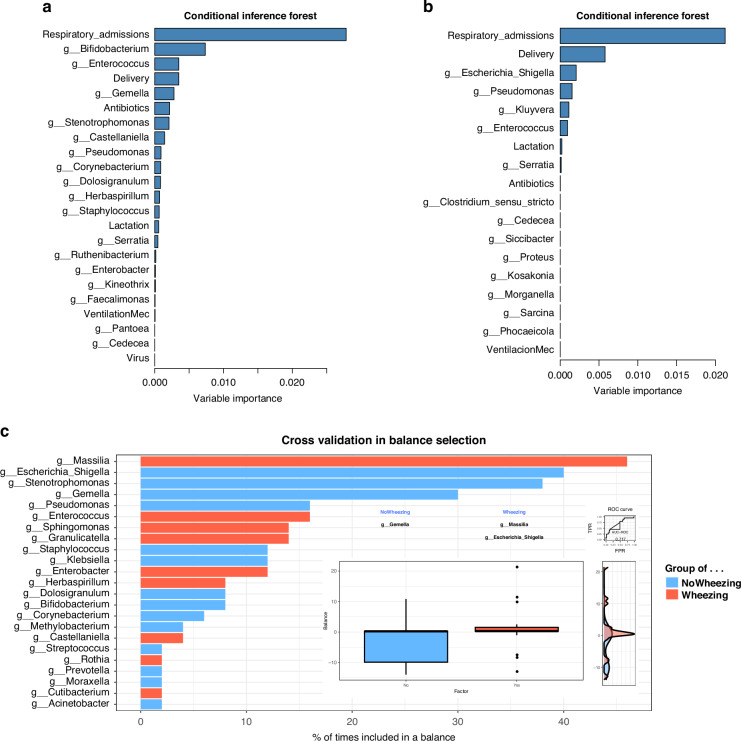
Prediction of recurrent wheezing development by integrating microbial and perinatal factors using Random Forest. Variable importance rankings for nasopharyngeal (**a**) and gut (**b**) models. **c** *Classification performance of the microbial balance model*. The boxplot represents the distribution of balance scores per group. On the right, the ROC curve and AUC (0.717) display the predictive performance of the model.

Random forest models demonstrated strong performance in distinguishing between wheezing and non-wheezing groups using microbiological parameters from NPAs and gut samples. The receiver operating characteristic (ROC) curves showed areas under the curve (AUC) of 0.812 for Model I (NPAs A vs. NPAs B) and 0.867 for Model II (Faeces A vs. Faeces B) (Fig. 7a;  7b), indicating robust predictive accuracy.

For NPAs, the most important predictors of wheezing development included during NICU admission, *Bifidobacterium*, *Enterococcus*, mode of delivery, *Gemella*, antibiotic use, *Stenotrophomonas*, *Castellaniella*, *Pseudomonas*, *Corynebacterium*, *Dolosigranullum*, *Staphylococcus*, breastfeeding, Serratia, Enterobacter, and mechanical ventilation, ranked in order of importance (Fig. 7a).

In contrast, for stool samples, key predictors associated with wheezing development included respiratory admissions, mode of delivery, *Escherichia/Shigella*, *Pseudomonas*, *Kluyvera*, *Enterococcus*, breastfeeding, *Serratia*, antibiotic use, *Clostridium sensu stricto*, *Cedecea*, *Siccibacter*, and mechanical ventilation during NICU admission, ranked by importance (Fig. 7b).

Using repeated 5-fold cross-validation, the selbal.cv() model identified a microbial balance that discriminated between children with and without wheezing. The model achieved an average cross-validated AUC of 0.717 (95% CI: 0.67–0.764), suggesting moderate discriminative ability. The final balance included *Gemella* as the genus most predictive of non-wheezing outcomes, whereas *Massilia* and *Escherichia/Shigella* were the strongest predictors of wheezing. The overall ROC curve, based on pooled predictions across all test folds, is shown in Fig. 7c, along with the taxa contributing to the balance and their relative importance.

## Discussion

This study highlights the factors influencing the nasopharyngeal and gut microbiota composition in preterm neonates and their potential association with the development of recurrent wheezing. Using advanced diversity metrics and multivariate analyses, we identified several clinical and environmental factors, such as gestational age, probiotic use, type of feeding, mode of delivery, and antibiotic treatment are known to influence both early microbial colonization and respiratory morbidity in preterm infants. In our analysis, we adjusted for these variables to account for potential confounding in the association between microbiota features and wheezing. Nevertheless, the interplay among these factors is complex: for example, extremely preterm infants are more likely to receive antibiotics and develop BPD, both of which can shape the microbiota. While our models aimed to control for these effects, residual confounding cannot be entirely excluded and should be explored in larger, stratified cohorts.

### Microbiota composition and clinical factors

We have adjusted for potential clinical covariates (including gestational age, BPD, and antibiotic use) in multivariable models. While antibiotic exposure remained significantly associated with wheezing, gestational age and BPD did not independently predict wheezing after adjustment, suggesting a more direct role of microbiota disruption. The nasopharyngeal microbiota, established within the first week of life, plays a key role in respiratory health by defending against pathogens and modulating immune responses. The presence of *Bifidobacterium* and *Staphylococcus* in non-wheezing infants suggests protective effects through immune modulation and pathogen inhibition.^60^ Consistent with prior work, early colonization by specific taxa has been linked to respiratory complications.^61,62^ In preterm neonates, altered microbiota, characterized by increased *Staphylococcus*, *Klebsiella*, and *Moraxella*, is associated with inflammation and increased wheezing risk.^60,63^ In contrast, protective commensals like *Corynebacterium* and *Dolosigranulum* are less abundant, which has been linked to asthma development in childhood.^60,64^

These imbalances may impair immune defence, increasing susceptibility to respiratory infections and inflammation.^65,66^ Pathogenic genera such as *Escherichia*/*Shigella*, *Serratia*, *Stenotrophomonas*, and *Haemophilus* are more common in wheezing infants, potentially priming a pro-inflammatory state, thereby predisposing neonates to recurrent respiratory morbidity.^67,68^ Genera like *Escherichia*/*Shigella* and *Stenotrophomonas*, more abundant in wheezing infants, may serve as dysbiosis markers consistent with associations between *Streptococcus* and *Staphylococcus*, and respiratory infections in infants.^22,69^ These taxa, along with *Moraxella* and *Haemophilus*, contribute to both acute and chronic respiratory morbidity. Gut-derived bacteria may also influence respiratory pathogenesis via the gut-lung immune axis.^13,70^

The gut microbiota regulates systemic inflammation and shapes respiratory outcomes through the gut-lung axis. In preterm infants, early dysbiosis, characterized by reduced microbial diversity and overgrowth of opportunistic pathogens like *Klebsiella*, *Escherichia*, and *Enterobacter*, is strongly associated with wheezing and respiratory diseases.^71^ Depletion of beneficial genera like *Lactobacillus* and *Bifidobacterium*, essential for immune system development and inflammation regulation, further highlights the impact of disrupted microbial homoeostasis.^72^ Microbial changes in the gut influence systemic inflammation and increase susceptibility to wheezing.^73^ For instance, studies by Fujimura et al,^74^ and Depner et al,^75^ demonstrated that early life colonization by beneficial gut microbes, such as *Lactobacillus* and *Bifidobacterium*, is associated with reduced asthma and wheezing risk in later childhood, while overgrowth of *Escherichia*/*Shigella* and *Klebsiella* increases.^70^ Gut microbiota shows more variability than nasopharyngeal microbiota, likely due to antibiotic exposure and formula feeding.^76,77^ Our study revealed that the gut microbiota of infants with BPD exhibited lower richness and diversity. However, this association is likely bidirectional: on the one hand, BPD-related clinical factors (such as inflammation, prolonged hospitalization, and antibiotic use) can disrupt microbial colonization; on the other hand, early dysbiosis may contribute to immune dysregulation and systemic inflammation, potentially exacerbating BPD severity. Therefore, while a clear causal direction cannot be established in this study, the link between microbial imbalance and BPD warrants further longitudinal and mechanistic investigation. The predominance of *Fusobacterium* and *Vagococcus* in extremely preterm infants (<28 weeks) suggests early dysbiosis, increasing risk of systemic inflammation, respiratory issues, and wheezing,^78^ aligning with prior research, linking gut microbiota dysbiosis to the development of wheezing.^63^

The early-life microbiome in the respiratory and gastrointestinal tracts is critical for immune development and long-term respiratory health outcomes in infants. This multicenter study examined the microbiota of premature newborns during the first week of life and its association with respiratory morbidity during their first year, underscoring its predictive value for early respiratory diseases.

In preterm infants, microbiota composition is influenced by clinical and environmental factors, including delivery mode, antibiotics, and breastfeeding. Neonatal antibiotic use, observed in 53.8% of cases, was linked to increased wheezing risk, likely due to disruption of both intestinal and nasopharyngeal microbiota.^79,80^ Antibiotics reduced microbial diversity and favoured the emergence of pathogenic taxa, such as *Klebsiella* and *Enterococcus*, both associated with an increased risk of wheezing in our study^81,82^ and with *Pseudomonas*, which are linked to wheezing.^83,84^ While antibiotics are essential for managing neonatal infections, they can disrupt microbial communities and increase susceptibility to conditions BPD and wheezing.^85^ To address potential confounding, our multivariate models assessing the relationship between microbiota and wheezing were adjusted for key clinical covariates, including gestational age, BPD, and antibiotic exposure (timing and duration), to better isolate the independent contribution of microbiota disruption to respiratory outcomes.

Alpha diversity analyses revealed significant associations between microbial richness and factors such as gestational age, probiotic administration, and breastfeeding. Probiotics effectively enhanced microbial diversity and stability, offering protective benefits in preterm neonates.^86^ Sex-specific differences in microbial profiles were also observed, supporting previous studies of distinct microbiota patterns between male and female neonates.^87^ Beta diversity analyses identified antibiotic exposure and gestational age as major drivers of microbial shifts, with antibiotics reducing diversity and promoting pathogenic genera.^88^ Delivery mode significantly influenced nasopharyngeal microbiota (R² = 0.0794, *P* = 0.049), while breastfeeding and mechanical ventilation were key determinants of gut microbiota (R² = 0.265, *P* = 0.001; R² = 0.224, *P* = 0.001). These findings align with evidence that caesarean delivery disrupts maternal microbiota transmission, hindering colonization by beneficial species and favouring pathogens like *Klebsiella* and *Enterococcus*.^87^

Predictive modelling using Random Forest accurately identified recurrent wheezing risk based on microbiome data, highlighted several key predictors of wheezing, including breastfeeding, mechanical ventilation, and antibiotic use for NPA samples, and respiratory admissions and antibiotic use for gut samples. Key genera like *Stenotrophomonas*, *Escherichia*/*Shigella*, and *Klebsiella* emerged as strong predictors, aligning with prior studies that identified these taxa as biomarkers of dysbiosis and respiratory complications.^89^ These results emphasize the potential of microbiota as both a risk marker and a target for preventive interventions.^90^ Early microbiome profiling in the NICU could serve as a non-invasive strategy to identify high-risk of developing recurrent wheezing and other respiratory morbidities. This predictive model, if validated externally, could support early identification of infants at risk for wheezing based on microbiota profiles. Early-life microbiota profiling in the NICU could be implemented as a non-invasive risk stratification tool. Infants identified as high-risk could benefit from enhanced monitoring, parental counselling, and potentially preventive interventions such as targeted probiotics, optimized antibiotic stewardship, or nutritional strategies to support microbiome development. For clinical translation, however, several steps remain necessary: external validation in larger, diverse cohorts; integration of microbiome data with other clinical predictors (e.g., biomarkers, genetic risk); and prospective trials to assess whether early intervention based on microbiome risk stratification improves respiratory outcomes. These steps are essential to move from biomarker discovery to actionable clinical practice. Integration into neonatal monitoring systems could guide preventive interventions or personalized follow-up.

### Strengths, limitations and future directions

A major strength of this study lies in the integration of multiple clinical and microbiological factors using advanced statistical tools, such as random forest and RDA, to elucidate the complex interplay between microbiota and preterm health outcomes. However, this study has several limitations, including a relatively small sample size and the lack of longitudinal data to track changes in microbiota composition over time. Moreover, the absence of functional microbiome analysis limits our ability to explore the molecular mechanisms behind microbiota-host interactions. Future research should incorporate multi-omics approaches, such as metagenomics, transcriptomics, and metabolomics, to investigate the functional roles of specific microbial taxa in respiratory health and disease.

Longitudinal studies with larger cohorts are necessary to validate our findings and determine causal relationships between microbiota and respiratory diseases, such as asthma,^59,91^ and could validate these findings and explore targeted interventions, such as probiotics, to modulate the microbiota in preterm infants. Furthermore, investigating how genetic predisposition interacts with microbial composition could offer valuable insights for developing personalized strategies for preventing recurrent wheezing and asthma. Another important direction for future research is exploring the mechanistic pathways linking the gut-lung axis and immune modulation, to deepen our understanding of the microbiome’s impact on respiratory health.

### Clinical implications and future research

Both the nasopharyngeal and gut microbiota demonstrate significant associations with respiratory health outcomes in preterm infants. Protective genera, such as *Corynebacterium* and *Bifidobacterium*, are consistently linked to reduced inflammation and improved respiratory outcomes, while the enrichment of opportunistic pathogens underscores the role of early life dysbiosis in respiratory disease pathogenesis. Clinical factors, including mode of delivery, antibiotic exposure, and breastfeeding, emerge as crucial determinants of microbial composition, influencing long-term health trajectories.^88^ These interventions refer to clinical strategies such as enhanced breastfeeding support, judicious use of antibiotics to avoid unnecessary disruption of the microbiota, and the administration of targeted probiotics aimed at promoting colonization by beneficial genera like *Bifidobacterium* and *Lactobacillus*. Such approaches could help preserve microbial diversity and reduce the risk of respiratory complications in preterm infants.^84^

Future research should prioritize interventions aimed at preserving microbial diversity and promoting colonization by beneficial genera, such as the use of probiotics, targeted nutritional strategies, and minimizing unnecessary antibiotic use. Such measures could mitigate the risk of respiratory complications in this vulnerable population, providing a foundation for improved neonatal care.

## Conclusions

In conclusion, the microbiota of preterm neonates during the first week of life plays a pivotal role in determining the risk of respiratory diseases, such as wheezing, later in life. Dysbiosis during this critical period, especially in the gut and nasopharyngeal microbiota, increases the likelihood of developing wheezing and asthma by one year of age. Clinical factors such as antibiotic use, delivery mode, and breastfeeding have a profound impact on microbiota composition, with specific genera such as *Moraxella*, *Corynebacterium*, and *Bifidobacterium* emerging as key biomarkers in this population, making them important targets for interventions to promote long-term respiratory health in preterm infants. These findings underscore the importance of preserving and modulating the microbiota during early life to prevent long-term respiratory complications, offering a foundation for future research and potential therapeutic interventions. Specific genera within the nasopharyngeal and gut microbiota were associated with increased or decreased risks of wheezing, suggesting that early-life interventions targeting microbial composition, such as tailored probiotics or optimized antibiotic stewardship, could potentially reduce respiratory morbidity in this vulnerable population. Preserving microbial diversity through appropriate clinical practices and interventions may help reduce the burden of wheezing and other respiratory diseases in this vulnerable population. Future research should focus on longitudinal analyses and mechanistic studies to explore the causal pathways linking microbiota composition to clinical outcomes and the development of microbiota-targeted interventions.

## Supplementary information

supplementary information

## Data Availability

The data are publicly available from the original study: European Nucleotide Archive accession number PRJEB88179.
