# Supplementary material for: Factors influencing preterm infant microbiota and their role in wheezing development
Source: Pediatr Res. 2025 Nov 15;99(7):2694–707. doi: 10.1038/s41390-025-04569-x (PMC13485686; doi:10.1038/s41390-025-04569-x)
Supplement: Supplementary file 1 [file 41390_2025_4569_MOESM1_ESM.pdf]

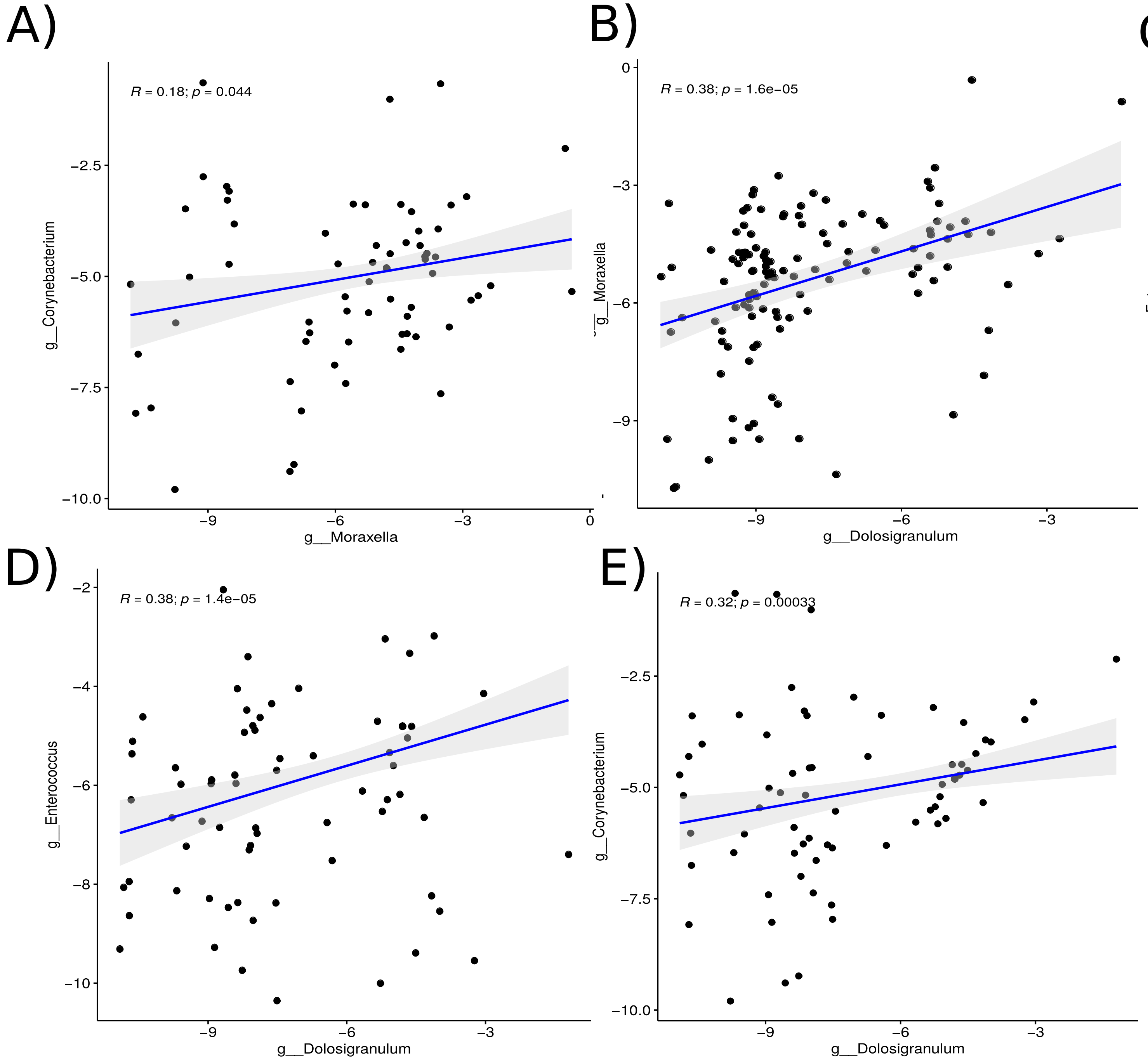
A)
R = 0.18; p = 0.044
g__Corynebacterium
g__Moraxella
B)
R = 0.38; p = 1.6e-05
g__Moraxella
g__Dolosigranulum
C)
R = 0.47; p = 5.8e-08
g__Enterococcus
g__Bifidobacterium
D)
R = 0.38; p = 1.4e-05
g__Enterococcus
g__Dolosigranulum
E)
R = 0.32; p = 0.00033
g__Corynebacterium
g__Dolosigranulum
